# From Neurovascular Compression to Neural Hyperexcitability: Integrating Microanatomy, Electrophysiology, and Computational Neuroscience to Understand Trigeminal Neuralgia and Hemifacial Spasm

**DOI:** 10.3390/cells15161437

**Published:** 2026-08-10

**Authors:** Hironori Okuhata, Masanori Aihara, Soichi Oya, Ryozo Nagai, Kenichi Aizawa

**Affiliations:** 1 Department of Translational Research, Clinical Research Center, Jichi Medical University Hospital, Shimotsuke 329-0498, Japan; 2 School of Medicine, Faculty of Medicine, Gunma University, Maebashi 371-8511, Japan; 3 Department of Neurosurgery, Gunma University Graduate School of Medicine, Maebashi 371-8511, Japan; 4 Jichi Medical University, Shimotsuke 329-0498, Japan

**Keywords:** neurovascular compression syndromes, trigeminal neuralgia, hemifacial spasm, ephaptic transmission, demyelination, electrophysiology, computational neuroscience, cable theory, ion channel dysfunction, microvascular decompression

## Abstract

**Highlights:**

**What are the main findings?**

- An integrative pathophysiological model based on microanatomy, electrophysiology, and computational simulations clarifies the relationship between neurovascular compression and abnormal neural activity.
- Pathophysiological concepts explain clinical symptoms and therapeutic responses in trigeminal neuralgia and hemifacial spasm.

**What are the implications of the main findings?**

- Biophysical and computational models provide quantitative insights into therapeutic mechanisms of microvascular decompression.
- Pathophysiological insights reveal inter-disease and case-to-case diversity in therapeutic responsiveness.

**Abstract:**

Neurovascular compression syndromes (NVCS), including trigeminal neuralgia (TN) and hemifacial spasm (HFS), are characterized by disabling symptoms caused by vascular compression of cranial nerves. Although microvascular decompression is an established treatment, mechanisms linking neurovascular compression to abnormal neural activity remain incompletely understood. In this review, we integrate evidence from microanatomical, electrophysiological, and computational studies to provide a mechanistic framework for NVCS. Chronic vascular compression induces focal demyelination, redistribution of voltage-gated ion channels, ectopic impulse generation, and ephaptic transmission, leading to abnormal neuronal excitation. We further summarize emerging evidence that persistent peripheral hyperactivity may contribute to electrophysiological alterations in central neural circuits. Particular attention is given to computational approaches, including cable theory and axonal interaction models, which offer quantitative insights into abnormal synchronization and cross-excitation among nerve fibers. Recent findings regarding ion channel dysfunction, including familial TN associated with gain-of-function calcium channel variants, are also discussed. Collectively, these findings support an integrated model linking neurovascular compression to clinical manifestations, and highlight the value of combining electrophysiology and computational neuroscience to improve mechanistic understanding and to guide future therapeutic strategies for NVCS.

## 1. Introduction

Neurovascular compression syndromes (NVCS) are pathological conditions in which cranial nerves are compressed by adjacent blood vessels, as in trigeminal neuralgia (TN) and hemifacial spasm (HFS) [1]. While these diseases exhibit distinct clinical features, depending on the affected nerve, the trigeminal nerve in TN and the facial nerve in HFS, they share a common pathogenesis of mechanical stress caused by vascular compression. TN is a paroxysmal, chronic pain disorder characterized by severe, stabbing pain in the area innervated by the trigeminal nerve [2]. HFS is characterized by involuntary contractions of facial muscles innervated by the unilateral facial nerve [3]. In 1934, Walter Dandy first identified this structural feature in TN patients, in whom the trigeminal nerve is compressed by a vessel or tumor [4,5]. Building on this concept, Peter Jannetta pioneered surgical interventions for NCVS during the 1960s and 1970s. By the 1990s, he successfully established microvascular decompression (MVD) techniques utilizing intraoperative microscopy [4,6].

While previous reviews have predominantly focused on clinical manifestations and surgical outcomes of NVCS, this review offers a different perspective by integrating neuronal microanatomy, electrophysiology, and mathematical modeling. By bridging the gap between macroscopic vascular compression and microscopic biophysical alterations, we provide a comprehensive framework that helps clarify the mechanistic underpinnings of TN and HFS. Although NVCS encompasses several cranial nerve disorders, this review specifically focuses on TN and HFS. This is because the microstructural and electrophysiological literature is most extensive for these two conditions, providing a framework to understand the biophysical core of the syndrome. To identify relevant articles, a comprehensive literature search was conducted in PubMed and Google Scholar for articles published up to July 2026. Search terms included those listed in the keywords, where “trigeminal neuralgia”, “hemifacial spasm”, “ephaptic transmission”, “demyelination”, and “microvascular decompression” are mainly used to research literature. Only peer-reviewed articles written in English were included, while abstracts, conference proceedings, and dissertations were excluded.

## 2. Vulnerability of the Transitional Zone

Lesions responsible for NVCS occur at the transitional zone separating central nervous system (CNS) segments and peripheral nervous system (PNS) segments of cranial nerves [7]. The cranial nerve possesses a transitional zone of myelination known as the Obersteiner–Redlich zone [7]. The central segment is myelinated by oligodendrocytes, but the peripheral segment is myelinated by Schwann cells [8]. This distinct microanatomical transition may explain its vulnerability to compression by adjacent blood vessels. The difference in embryological origin leads to a structural disparity in that the PNS segment is more resilient to compression than the CNS segment [9]. Consequently, the transitional zone, which comprises various types of myelination, represents a junction vulnerable to shear stress [7,9]. The transitional zone of the facial nerve is short, measuring 2.5 mm [10]. Consequently, HFS may often be triggered by vascular compression at the proximal nerve root [10]. In contrast, the transitional zone of the trigeminal nerve is longer, extending 4 mm [10], which may explain why TN tends to be triggered by vascular compression in the distal cisternal portion [11].

Furthermore, nerve fibers in the CNS segment lack the endoneurium, perineurium, and epineurium [12]. The structural barrier in the nerve root is a glial limiting membrane, referred to as the glial sheath, in which astrocyte foot processes surround the central myelin, analogous to the epineural sheath in PNS [12]. Although this glial sheath acts as a barrier against vascular compression, the sheath of the facial nerve becomes thinner in the distal portion [12]. Thus, thinning of the neural sheath in the nerve root leaves the cranial nerve sensitive to vascular compression, resulting in damage to nerve fibers.

## 3. Vascular Compression-Induced Nerve Degeneration

Mechanical compression of the structural weak points of cranial nerves by adjacent blood vessels triggers various biological phenomena. There is a hypothesis that pulsatile vascular compression causes bioresonance matching the natural frequency around the trigeminal nerve, thereby damaging the nerve fibers [13]. This bioresonance hypothesis potentially accounts for the clinical heterogeneity of neurovascular compression, in which mechanical contact does not universally manifest as clinical symptoms.

Microstructural changes in nerve fibers occur at the site of vascular compression. Biopsy specimens of the trigeminal nerve root obtained from TN patients have revealed axonal loss, focal demyelination with residual myelin debris, dense packing of demyelinated axons, and a reduction in intervening astrocytic processes [14,15]. These pathological alterations, specifically axonal injury, demyelination, and axonal crowding, alter electrical excitability of the nerve, thereby contributing to ectopic firing and ephaptic transmission [14,15], which form the pathophysiological basis of NVCS.

Under the International Classification of Headache Disorders-3, TN is broadly categorized into classical and idiopathic TN [16]. While classical TN is characterized by neurovascular compression with morphological changes, these features are absent in idiopathic TN [16]. The latter category comprises patients experiencing pain in the trigeminal region despite the absence of identifiable mechanical vascular compression [17]. However, recent studies have reported cases of idiopathic TN involving non-vascular pathogenic intraoperative findings [18,19]. These include angulation or torsion of the root axis caused by arachnoid thickening or granulomatous adhesion [18], as well as a fibrous ring encircling the nerve [19]. Surgical intervention targeting these structures successfully decompresses the trigeminal nerve and alleviates clinical symptoms [18,19]. Furthermore, abnormal cerebrospinal fluid flow has been detected in idiopathic TN, which can be resolved via arachnoid dissection [20]. These findings suggest that idiopathic TN can arise from previously unrecognized pathogenic causes independent of vascular compression.

## 4. Ion Channel Dynamics and Ectopic Firing

### 4.1. Voltage-Gated Channels

Nerve degeneration alters axonal electrophysiological properties by disrupting ion channel dynamics, which consequently trigger ectopic firing. Considering that multiple sclerosis (MS) involves neurodegeneration with demyelination in the CNS and is sometimes responsible for TN, we believe that this disease could be considered as an extrapolated model of sodium channel redistribution in demyelinated axons in TN and HFS, although such redistribution has not been directly demonstrated at the trigeminal or facial nerve compression site. To restore impaired neurotransmission, neurons upregulate channel expression [21], adaptively increasing the total density of sodium channels on the plasma membrane in MS [22]. Specifically, voltage-gated sodium channels (Nav), Nav1.2 and Nav1.6 relocate and redistribute diffusely across the demyelinated membrane in MS [23]. While Nav1.6 is the predominant Nav in mature neurons, normally clustering at nodes of Ranvier to facilitate saltatory conduction [24], Nav1.2 is typically expressed diffusely along unmyelinated axons during early development [25].

Furthermore, demyelination exposes voltage-gated potassium channels (Kv), triggering abnormal leakage of potassium current [26], which consequently elevates the extracellular potassium concentration around axons [27]. The relationship between trans-membrane ion gradients and equilibrium potentials is governed by the Nernst equation:

(1) $$E_{K}=\frac{RT}{zF}\log_{e}\frac{{\left[K^{+}\right]}_{out}}{{\left[K^{+}\right]}_{in}}$$

where $E_{K}$ represents the potassium equilibrium potential, R represents the gas constant, T represents absolute temperature, z represents charge (z=1 for potassium), F represents the Faraday constant, and e represents Euler’s constant. According to this relationship, a rise in extracellular potassium concentration (${\left[K^{+}\right]}_{out}$) elevates the potassium equilibrium potential ($E_{K}$). This elevation depolarizes the resting membrane potential and decreases the outward potassium driving force (${V_{m}-E}_{K}$), ultimately provoking ectopic axonal firing [27]. The aforementioned, spontaneous depolarization rooted in demyelination, resting membrane potential depolarization, and a decreased firing threshold have been experimentally confirmed in the idTNC model, an intradural compression model of the trigeminal nerve root [28].

Some cases of TN without vascular compression suggest that channelopathy may contribute to familial TN [29]. Through whole-exome sequencing of these patients, rare variants have been identified in the *CACNA1H* gene, which encodes Cav3.2, a type of voltage-gated T-type calcium channel [29]. Electrophysiological analysis was performed by expressing these Cav3.2 variants in human embryonic kidney (tsA-201) cells [29]. In several variants, the activation threshold shifted in the hyperpolarizing direction, resulting in enhanced inward calcium current around the resting membrane potential [29]. Furthermore, in certain other variants, the time constant for recovery from inactivation was shortened [29]. Using experimental data from these gain-of-function variants, computer simulations of firing in thalamic reticular neurons, which form part of the trigeminal pathway, predicted a reduced threshold and an increased frequency for transient, sustained abnormal firing [29]. These findings suggest that neural pathway hyperexcitability, driven by channelopathy, may contribute to the pathogenesis of TN, particularly in cases with concomitant continuous pain.

### 4.2. Mechanosensitive Channels

Recent evidence from rat TN models suggests a potential role of mechanosensitive Piezo2 channels, which are heavily modulated by neuroinflammatory cascades driven by vascular compression in TN [30,31]. Mechanical stress triggers the release of adenosine triphosphate from damaged axons and myelin sheaths, which subsequently binds to P2X and P2Y receptors on trigeminal neurons, elevating the intracellular Ca^2+^ concentration [30]. This Ca^2+^ influx drives protein kinase C and mitogen-activated protein kinase signaling pathways, which transcriptionally upregulate Piezo2 channels, as well as nociceptive peptides such as calcitonin gene-related peptide and substance P [30]. Once released, these peptides bind to receptors on peripheral Merkel cells, increasing intracellular cyclic adenosine monophosphate and hypersensitizing Merkel-cell Piezo2 channels [30]. Concurrently, vascular compression elevates expression of interleukin-6 (IL-6) in the microenvironment [31]. In concert with this IL-6 upregulation, Piezo2 is excessively expressed in nociceptive C fibers [31]. Consequently, this dual mechanism of sensitization of low-threshold mechanoreceptors and high expression of Piezo2 in pain pathways allows Piezo2 to be directly triggered by innocuous facial contact as well as by chronic arterial pulsation, generating paroxysmal pain [30,31].

## 5. Ephaptic Transmission and Its Clinical Manifestations

### 5.1. Mechanism of Ephaptic Transmission Induced by Demyelination

Ectopic excitation propagates along demyelinated nerve fibers, generating abnormal signals, and non-synaptic nerve communication occurs in unmyelinated regions of axons [24]. Myelin functions as electrical insulation, permitting signal transmission without interference. However, ephaptic transmission (electrical crosstalk) occurs between axons that lack myelin insulation [32,33]. As noted in Section 3, demyelinated axons are densely packed, and current leakage from insufficiently insulated areas causes excitatory interactions between adjacent axons [15,33].

### 5.2. Mathematical Modeling Based on Cable Theory

Mathematical models can be constructed based on cable theory [34] to describe interactions between axons resulting from ephaptic transmission [33]. For simplicity, assuming two parallel nerve fibers with identical diameters, their membrane potentials, $v_{m1}$ and $v_{m2}$, can be expressed by the following system of linear partial differential equations with respect to distance x and time t:

(2) $$\left\{\begin{array}{c} \gamma \frac{\partial^{2}v_{m1}}{\partial x^{2}}-\alpha \frac{\partial^{2}v_{m2}}{\partial x^{2}}=c\frac{\partial v_{m1}}{\partial t}+j_{ion,\;1}-I_{1}\\ \gamma \frac{\partial^{2}v_{m2}}{\partial x^{2}}-\alpha \frac{\partial^{2}v_{m1}}{\partial x^{2}}=c\frac{\partial v_{m2}}{\partial t}+j_{ion,\;2}-I_{2} \end{array}\right.$$

where the coupling coefficients γ and α are defined as follows:

(3) $$\gamma =\frac{r_{a}+r_{e}}{2r_{a}r_{e}+{r_{a}}^{2}},\;\alpha =\frac{r_{e}}{2r_{a}r_{e}+{r_{a}}^{2}}$$

Here, $r_{a}$ represents the axoplasmic resistance per unit length, $r_{e}$ is the extracellular space resistance per unit length, c is the axonal membrane capacitance per unit length, $j_{ion}$ denotes the active ionic current, and I is the external applied current. The second term on the left-hand side of Equation (2) indicates the mutual interaction between two nerve fibers. Demyelination causes axons to pack closely together, increasing $r_{e}$ and consequently increasing α, the parameter representing the coupling strength.

### 5.3. Computer Simulation: Conduction in Bundles of Demyelinated Fibers

The mathematical model was extended to accommodate a large number of parallel nerve fibers, and computer simulations effectively illustrated potential mechanisms of the synchronization phenomena of conduction and the recovery of excitation [35]. The model is formulated based on simplified anatomical and electrical assumptions, including perfectly parallel, axisymmetric, and identically sized fibers with nodal ion channels and passive myelin sheaths [35].

Based on these assumptions, in a nerve bundle consisting of N parallel axons, the membrane equation for the k-th fiber in the myelin-sheathed region is described by the following partial differential equation with respect to distance x and time t:

(4) $$c_{m,k}(x)\frac{\partial V_{k}}{\partial t}=\frac{1}{r_{f}}\frac{\partial^{2}V_{k}}{\partial x^{2}}+\frac{\partial I_{eph}(V_{1},\;\ldots ,\;V_{N})}{\partial x}-g_{m,\;k}(x)V_{k}$$

where $V_{k}$ is the membrane potential, $c_{m,k}$ is the capacitance per unit length of the myelin sheath, $g_{m,\;k}$ is the leakage conductance per unit length of the myelin sheath, $r_{f}$ is the electrical resistance per unit length of the axoplasm, and $I_{eph}$ is the ephaptic current between the fibers. The membrane potential $V_{k}$ is defined as the difference between the intracellular potential $\phi_{k}$ and the extracellular potential $\phi_{0}$ in the nerve bundle as follows:

(5) $$V_{k}(x,\;t)=\phi_{k}(x,\;t)-\phi_{0}(x,\;t)$$

The ephaptic current is derived from the current balance in the nerve bundle and Ohm’s law in the axial direction, given by:

(6) Ieph(V1, …, VN)=−1rfr0rf1+N·r0rf∑n=1N∂Vn∂x

where $r_{0}$ is the electrical resistance per unit length of the extracellular space inside the nerve bundle. The following Equation (7) is obtained by integrating Equations (4) and (6), and for N=2, it essentially corresponds to Section 5.2:

(7) $$\frac{r_{f}+(N-1)r_{0}}{{r_{f}}^{2}+Nr_{f}r_{0}}\frac{\partial^{2}V_{k}}{\partial x^{2}}-\frac{r_{0}}{{r_{f}}^{2}+Nr_{f}r_{0}}\sum_{n\neq k}\frac{\partial^{2}V_{n}}{\partial x^{2}}=c_{m,k}(x)\frac{\partial V_{k}}{\partial t}+g_{m,\;k}(x)V_{k}$$

$\alpha_{N}:=\frac{r_{0}}{{r_{f}}^{2}+Nr_{f}r_{0}}$ represents the coupling strength.

Similarly, the membrane equation at the nodes of Ranvier is expressed as follows:

(8) $$c_{nd}\frac{\partial V_{k}}{\partial t}=\frac{1}{r_{f}}\frac{\partial^{2}V_{k}}{\partial x^{2}}+\frac{\partial I_{eph}(V_{1},\;\ldots ,\;V_{N})}{\partial x}-j_{ion,\;k}-j_{ex,\;k}$$

where $c_{nd}$ is the capacitance per unit length at the node, $j_{ion,\;k}$ is the active membrane ionic current per unit length, and $j_{ex,\;k}$ is the externally applied stimulus current per unit length. $j_{ion}$ depends on the Frankenhaeuser–Huxley model [36].

First, simulations were conducted on a single nerve fiber, in which conduction velocity decreased monotonically as the myelin sheath thinned due to demyelination [35]. Furthermore, while conduction velocity only decreased 13% when the myelin sheath thickness was reduced to 20% of its normal value, it dropped sharply beyond a critical threshold of 2%, resulting in axonal conduction block [35].

Next, simulations were performed on three nerve fibers to examine interactions in excitation conduction between fibers. When the three fibers were stimulated with a time delay of 0.5 ms each, a synchronization phenomenon was observed, in which the three action potentials aligned and propagated at exactly the same velocity after traveling a certain distance [35]. This synchronized conduction velocity was slower than the conduction velocity of a single nerve fiber under the same conditions [35]. When only two of the three fibers were stimulated with a time delay of 0.5 ms each, impulses of the two stimulated fibers synchronized, while a subthreshold wave was formed in the unstimulated fiber due to the ephaptic current [35]. As the synchronization formed a “coupled pulse”, the ephaptic current increased and elicited a “spurious spike” at a node of Ranvier in the unstimulated fiber, generating both an orthodromic and an antidromic spike [35]. The orthodromic spike rapidly synchronized with excitation of the stimulated fibers [35]. That is, the unstimulated fiber was recruited via ephaptic transmission [35].

Furthermore, simulations were performed on a nerve bundle consisting of 500 fibers, including demyelinated ones. The strength of the electrical coupling between the fibers in the bundle is defined by the coupling strength β: $\beta =\frac{\rho_{0}}{\rho_{ax}}$, where $\rho_{0}$ is the specific resistivity of the extracellular space inside the nerve bundle, and $\rho_{ax}$ is that of the axoplasm. The proportion of demyelinated fibers in the bundle was set to 25%, and the coupling strength was set to β=5. Simulation results revealed that although conduction blocks occurred at demyelinated sites along demyelinated fibers, these fibers were re-excited a few nodes downstream by the ephaptic current from the normal fibers, subsequently synchronizing with them [35]. This suggests that at a certain coupling strength, normal fibers might help restore conduction of demyelinated fibers [35].

While earlier multi-fiber models assumed uniform axonal diameters, more recent computational studies using large peripheral nerve bundles have introduced structural heterogeneity, revealing that wider distributions of axon diameters exert a dispersive effect that counteracts synchronization [37]. This implies that a higher degree of fiber heterogeneity requires significantly stronger ephaptic coupling to achieve full alignment of traveling action potentials [37].

Crucially, the present model simulated two pivotal phenomena: (1) synchronization of action potentials that elicits “spurious spikes” in unexcited fibers, and (2) an endogenous compensatory mechanism whereby intact fibers can restore conduction in adjacent demyelinated axons [35]. These findings may help to explain how non-nociceptive tactile stimuli cross over to pain pathways in TN (trigger zones) and how motor signals spread to adjacent facial muscle branches in HFS (lateral spread response) (See Section 5.5).

### 5.4. Quasi-Static Electric Dipole Model

As a complementary approach to the cable model, the quasi-static electric dipole model may be useful for understanding ephaptic transmission [38]. This model represents the electric field formed in the extracellular space by electric dipoles generated by the opening and closing of voltage-gated ion channels, which are densely packed at the nodes of Ranvier in myelinated nerves [38].

When Nav open at nodes of Ranvier, sodium ions rapidly flow into the intracellular space, driven by the electrochemical potential, forming a microscopic electric dipole across the cell membrane. The dipole moment of a single channel, p(t), is defined using the membrane thickness $d_{membrane}$ and the accumulated charge ∆q as follows:

(9) $$p(t)=d_{membrane}∆q\hat{k}$$

where $\hat{k}$ represents the unit vector with the direction of the dipole, pointing from extracellular to intracellular, perpendicular to the surface of the membrane. The accumulated charge ∆q is determined by the sodium current $I_{Na}$ flowing during a time interval ∆t, expressed as:

(10) $$∆q=I_{Na}∆t$$

The electric field E(r) generated by a single electric dipole at a position r is given by the following equation:

(11) $$E(r)=\frac{1}{4\pi \varepsilon_{0}\varepsilon_{r}}\frac{3\hat{r}(p(t)\cdot \hat{r})-p(t)}{r^{3}}$$

where $\varepsilon_{0}$ is vacuum permittivity, $\varepsilon_{r}$ is relative permittivity, $\hat{r}=\frac{r}{r}$, and $r=\left|r\right|$. At nodes of Ranvier, ion channels are densely packed in a ring-like formation. Consequently, quasi-static electric fields generated by these individual dipoles superimpose to form a powerful electric field. Therefore, the total electric field $E_{tot}(r)$ generated at node of Ranvier is the summation of fields from all ion channels:

(12) $$E_{tot}(r)=\sum_{ionchannel}\frac{1}{4\pi \varepsilon_{0}\varepsilon_{r}}\frac{3\hat{r}(p(t)\cdot \hat{r})-p(t)}{r^{3}}$$

The induced membrane potential $V_{m}$ generated across the cell membrane of an adjacent axon by this total electric field is defined as follows:

(13) $$V_{m}=-\int_{membrane}E_{tot}(r)\cdot dr$$

Based on the proposed quasi-static electric dipole model, several physiological and pathological characteristics of nerve conduction can be conceptually interpreted. First, in a single isolated axon, the induced extracellular electric field generated at a specific node of Ranvier attenuates rapidly with distance, which is insufficient to depolarize the next downstream node beyond the threshold level across the internodal distance [38]. However, when multiple myelinated nerve fibers run in parallel, the induced electric field can effectively depolarize nodes of Ranvier of adjacent quiescent fibers [38]. Consequently, excitation is predicted to propagate not via a purely linear path in a single fiber, but rather via a “zigzag” pattern mediated by inter-axonal ephaptic transmission [38]. This mechanism highlights a distinction between myelinated and unmyelinated axons. While high-density nodal ion channels in myelinated nerves form a strong, localized total electric field $E_{tot}(r)$ that enables efficient ephaptic transmission, their sparse distribution in unmyelinated nerves creates a weak field [38].

Furthermore, this model provides an electrodynamic explanation for the dual pathology of demyelinating diseases. Disruption of nodal structure reduces channel density, weakening the induced field and leading to conduction block. Conversely, the resulting severe axonal crowding drastically reduces the inter-axonal distance r, which significantly amplifies the electric field, allowing a single active node to depolarize nodes of multiple surrounding silent fibers, potentially triggering abnormal cross-talk known as ectopic firing. However, as this electric dipole model is not experimentally established, it should be applied cautiously to pathophysiology. The methodological features, key findings, and clinical implication of these mathematical models are summarized in Table 1.

### 5.5. Clinical Applications to Trigeminal Neuralgia and Hemifacial Spasm

Ephaptic transmission accounts for several clinical features of NVCS. For instance, it may explain the existence of trigger points in trigeminal neuralgia. A trigger point is a location where a mild sensory stimulus induces trigeminal neuralgia, and it is mainly reported in the oral and nasal regions [39]. In the root of the trigeminal nerve, tactile sensation fibers and pain sensation fibers run in close proximity [40]. Under normal conditions, these two pathways remain independent; however, demyelination caused by vascular compression facilitates ephaptic transmission between these fibers [40]. Trigger zones occur predominantly in oral and peri-oral regions because these areas have a dense distribution of large Aβ and Aδ fibers that run in a superior position in the nerve root, making them uniquely vulnerable to compression by the superior cerebellar artery [41]. Consequently, tactile stimulation applied to a trigger point induces trigeminal neuralgia through this ephaptic transmission between tactile and nociceptive fibers. This is highly consistent with recent pathophysiological evidence indicating that sensory root demyelination is sufficient to fundamentally transform non-nociceptive touch into paroxysmal pain pathways [42].

**Figure 1:**
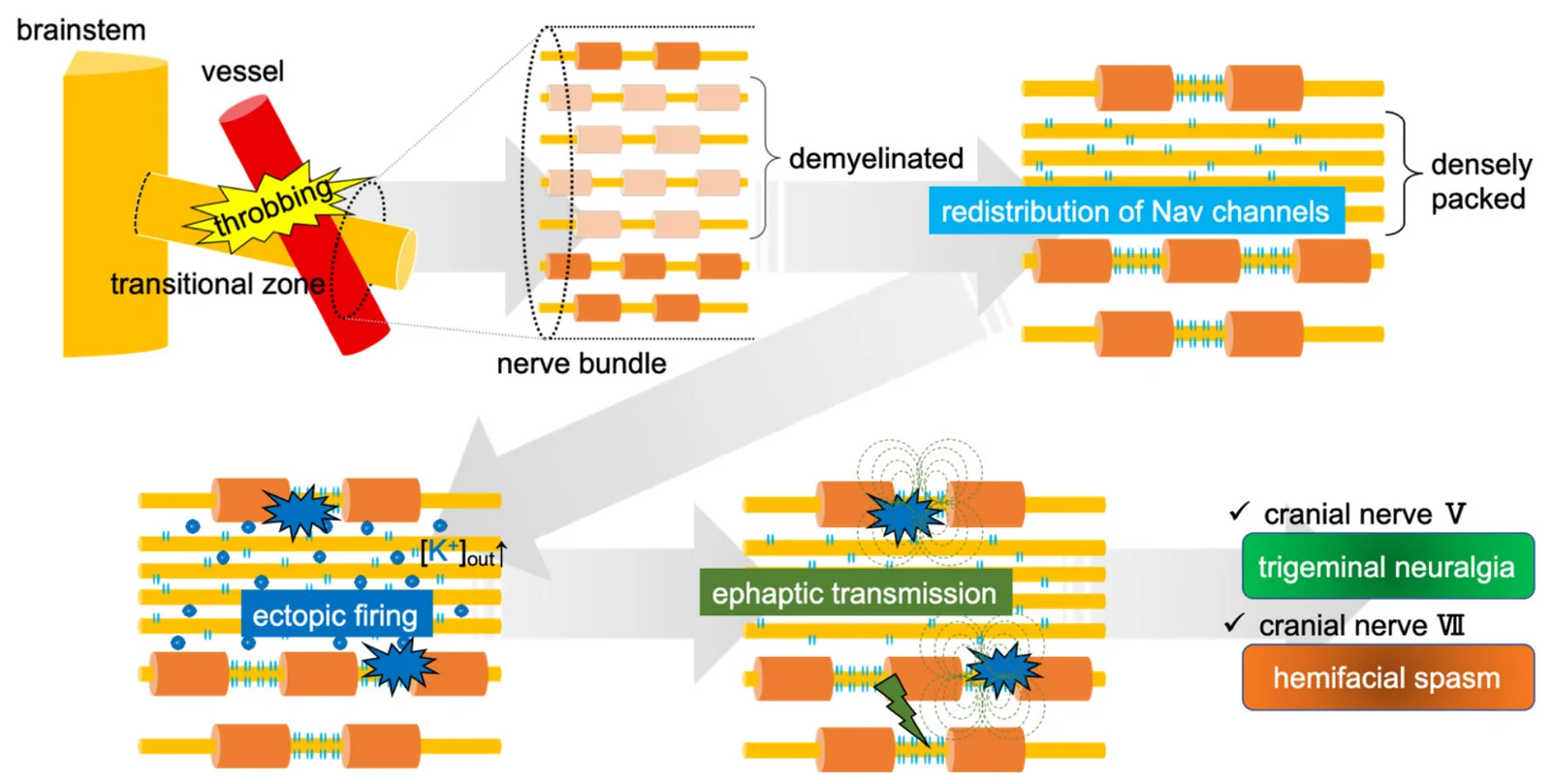
Proposed electrophysiological concept of demyelinated fibers in TN and HFS. Vascular compression in the transitional zone induces demyelination of cranial nerves. The demyelinated area in the nerve fiber bundle consists of densely packed axons with redistributed Nav. These demyelinated axons also release potassium ions into the extracellular matrix, triggering ectopic firing. Furthermore, high-density Nav clusters at nodes of Ranvier generate induced potentials that propagate to adjacent fibers, thereby causing ephaptic transmission. These electrophysiological phenomena eventually manifest as clinical symptoms characteristic of trigeminal neuralgia or hemifacial spasm.

Furthermore, ephaptic transmission underlies the lateral spread response (LSR) in HFS. LSR is a phenomenon in which stimulation of one branch of the facial nerve induces contraction of facial muscles innervated by other branches [43]. Excitation transmitted antidromically from the stimulated facial nerve branch propagates to other motor branches through ephaptic transmission [43]. Electrophysiological evidence in human subjects supports this mechanism, showing that LSR originates from ephaptic transmission at the vascular compression site [44]. Furthermore, a central mechanism in the facial nucleus induced by peripheral hyperexcitation is also reportedly involved in LSR (See Section 6). The overall proposed electrophysiological mechanism from vascular compression to clinical symptoms in TN or HFS is illustrated in Figure 1.

## 6. Central Electrophysiological Alterations Induced by Chronic Peripheral Discharges

While the preceding sections have focused on peripheral mechanisms, accumulating evidence indicates that persistent abnormal peripheral activity also induces electrophysiological changes in the central nervous system. This section summarizes current evidence for these central mechanisms in TN and HFS.

Nociceptive inputs I in TN are integrated and perceived as severe paroxysmal pain through the ascending pathway, consisting of the trigeminal ganglion, brainstem, thalamus, and primary somatosensory cortex (S1) [45]. Recent computational approaches have successfully formulated these central nociceptive pathways using Hodgkin–Huxley models [45]. Dynamical system analysis reveals that the response of S1 neurons to the mean intensity of noxious stimuli $I_{0}$ undergoes a transition governed by a supercritical Hopf bifurcation [45]. In a specific range of $I_{0}$, membrane potential dynamics of S1 shift into a stable limit cycle, which may correspond to the burst firing observed in clinical settings [45]. Furthermore, numerical simulations modulating conductance of Nav1.8 $g_{NaS}$, a key channel implicated in hyperalgesia, suggest that alterations in this conductance directly modulate the amplitude of the S1 voltage response [45]. Moreover, these mathematical frameworks provide critical insights into modulation of pain via the thalamocortical loop [45]. Simulating effects of an external stimulus representing transcranial direct current stimulation ($I_{tDCS}$) over the primary motor cortex (M1) reveals its impact on S1 network dynamics [45]. Applying this external stimulus to M1 remotely activates the descending pain inhibitory system, subsequently suppressing hyperexcitable responses in S1 [45]. These simulation results suggest that direct electrical intervention targeting M1 may have the potential to “reset” the central nociceptive cognitive network, offering a promising theoretical foundation for optimized, non-invasive neuromodulation in refractory TN patients [45].

CNS mechanisms also contribute significantly to HFS. While Section 5 details the peripheral theory, peripheral chronic excitation induces plasticity in CNS and facial motor nucleus (FMN) hyperexcitability [46]. First, latency to elicit the LSR significantly exceeds the peripheral loop conduction time, implying a central synaptic delay in the FMN pathway [47]. Furthermore, blink reflexes elicited by paired-pulse stimulation reveal that the physiological suppression observed in healthy individuals is markedly diminished in HFS patients, suggesting a profound dysfunction of brainstem inhibitory interneurons [47]. Facial F-wave studies further support this, showing abnormally elevated duration and frequency on the symptomatic side, which reflects enhanced FMN motor neuron excitability [47]. In conclusion, chronic abnormal peripheral discharges propagate antidromically to the brainstem, driving long-term plastic alterations and subsequent FMN hyperexcitability [47].

Furthermore, deletion of the synkinetic response of blink reflex and LSR after botulinum toxin treatment suggests that this central hyperexcitability continuously stimulates the facial muscles, which in turn could trigger somatosensory afferent cutaneous impulses via the trigeminal nerve [48]. Traveling through the trigeminal ganglion and trigeminal nucleus, these impulses reach the contralateral somatosensory ventral posteromedial nucleus of the thalamus and the facial somatosensory cortex [48]. Consequent activation of this cortical area stimulates the motor and supplementary motor areas (the extrapyramidal and basal ganglia systems), ultimately reinforcing FMN motor neuron activity and perpetuating peripheral facial muscle stimulation [48].

## 7. Treatment and Its Responsiveness for Trigeminal Neuralgia and Hemifacial Spasm

Carbamazepine is the first-line drug for TN [49]. Oxcarbazepine is a keto-analog of carbamazepine that does not produce epoxide metabolites and that reduces side effects more effectively than carbamazepine [50]. Carbamazepine and oxcarbazepine act as Nav blockers and delay recovery of sodium currents from the inactivated state [50,51,52]. They alleviate pain by inhibiting ectopic firing of nociceptive fibers [49,50,53]. In HFS, although its blocking of facial nerve hyperexcitability has been noted, their therapeutic efficacy remains limited [54].

MVD is widely recognized as a definitive treatment for NVCS [55]. It is a surgical procedure that relieves compression by separating the offending vessel from the nerve [55]. In patients with HFS, successful decompression of the offending vessel via MVD often results in immediate disappearance of the abnormal muscle response (AMR) evoked by the LSR [56]. This intraoperative finding serves as a valuable indicator for assessing therapeutic efficacy [56]. However, the therapeutic response to MVD is not universally immediate or absolute. The estimated cure rate, 3 years post-surgery is reportedly 91.4% for TN and 93.6% for HFS, indicating that approximately 10% of patients achieve only incomplete symptom relief or fail to derive postoperative benefits [57]. Furthermore, a distinct chronological divergence in treatment responsiveness exists between TN and HFS. In TN, delayed resolution, characterized by a slow disappearance of symptoms following surgery, is extremely rare, occurring in only 3.3% of cases [58]. Additionally, the pain cessation rate immediately after surgery or in 3 months reaches 96%, underscoring that symptoms typically resolve at an early stage [59]. Conversely, HFS presents a highly diverse clinical course, encompassing cases with immediate postoperative symptom resolution, gradual disappearance, or transient re-exacerbation [60].

Symptomatic improvement after MVD is broadly classified into an immediate effect after surgery and delayed healing spanning several months, which may explain the case-specific variation in therapeutic responsiveness following MVD. The immediate effect is thought to be due to the prompt blockade of ephaptic transmission in the demyelinated region resulting from decompression of pulsatile stress caused by the offending vessel [61,62]. In the mathematical model discussed in Section 5, this mechanism corresponds to a reduction in coupling strength among densely packed axons. On the other hand, delayed healing is thought to be deeply involved in gradual structural remyelination [63]. Pathological examinations using electron microscopy on nerve roots of patients with TN have observed incomplete remyelination, in which newly formed thin myelin sheaths cover closely apposed demyelinated axons in the zone of vascular compression [63]. Once remyelination is completed after MVD, the electrical insulation between axons could be fully restored, further leading secondary to suppression of hyperexcitable central cranial nerve nuclei [64]. The significance of these remyelination processes is paradoxically supported by the fact that the long-term complete remission rate after MVD is significantly lower in TN patients with concomitant multiple sclerosis, a central demyelinating pathology [65].

The diversity among cases in these recovery processes correlates strongly with the preoperative severity of vascular compression [66,67]. Quantitative investigations using high-field magnetic resonance imaging with macromolecular proton fraction mapping techniques reveal that the myelin volume is significantly reduced in severe-compression TN cases accompanied by nerve deformation or indentation [66]. Interestingly, it has been reported that the long-term success rate of MVD in these cases with severe compression is significantly higher than in mild compression cases [67]. Therefore, if distinct and severe vascular compression can be fully resolved by MVD, long-term remission can be expected through subsequent remyelination.

Substantial case-to-case variation exists during the period required to achieve this long-term remission [68,69]. Identified factors involved in the time lag of delayed healing include the longer duration of preoperative illness, the severity of symptoms, and differences in disappearance patterns of intraoperative AMR in HFS cases [68]. These findings suggest that even if the physical blockade of ephaptic transmission is achieved intraoperatively by releasing chronic vascular compression, a corresponding amount of time is required until the final structural remyelination is completed. Therefore, considering these pathophysiological recovery processes, it is rational to expect a monitoring period of approximately 3–6 months for evaluating the surgical effect and predicting the prognosis of MVD [69].

Furthermore, this distinct chronological divergence in postoperative timelines between TN and HFS likely arises from the unique central neuroplastic alterations characteristic of each system, as discussed in Section 6. TN involves a pain-processing mechanism that is directly dependent on the nociceptive input I from the trigeminal nerve. Once MVD mechanically interrupts these peripheral ectopic inputs, the hyperexcitability of S1 may be immediately suppressed, leading to early symptom resolution. In contrast, HFS is governed by a self-amplifying loop mechanism wherein antidromic peripheral inputs trigger and reinforce motor neuron activity of FMN. Consequently, elimination of peripheral abnormal signals via MVD does not necessarily result in an immediate alleviation of symptoms, which may explain the delayed chronological course frequently observed in HFS cases.

Gamma Knife radiosurgery is a non-invasive radiotherapeutic option selected for drug-resistant TN [70,71]. It is a form of stereotactic radiosurgery that utilizes cobalt-60 (^60^Co) as its radiation source [72]. Its primary clinical indications include arteriovenous malformations, acoustic neuromas, metastatic brain tumors, and TN [72]. Targeting the trigeminal nerve root with a radiation dose of 70 to 90 Gy induces localized axonal degeneration and necrosis [73]. Pain relief takes an average of 1 month, indicating a delayed therapeutic onset [73].

Botulinum toxin type A therapy for HFS is considered a more immediate and non-invasive treatment than MVD [74]. Botulinum toxin blocks release of acetylcholine by interrupting fusion of synaptic vesicles with the cell membrane in presynaptic nerve terminals [74]. Acetylcholine is a neurotransmitter responsible for synaptic transmission [74]. Botulinum toxin injected directly into targeted muscles, including the orbicularis oculi, zygomaticus, orbicularis oris muscles, and depressor anguli oris muscle, is locally absorbed and relaxes excessively contracted muscles by preventing synaptic transmission from presynaptic nerve terminals to muscles for an average of approximately 15 weeks [74]. Recent studies suggest that botulinum toxin treatment is also a promising therapy for TN [75]. Meta analysis research reports that onabotulinumtoxinA or lanbotulinumtoxinA therapy is effective for pain intensity reduction with transient facial asymmetry as the main adverse event [75]. Given the TN pathophysiology discussed in Section 4.2, we suggest that afferent excitation initiated from Aδ and C fibers in the subcutaneous layer may be inhibited by botulinum treatment.

## 8. Conclusions

This review bridges macro-level vascular compression with clinical features of NVCS by exploring integrative pathophysiology encompassing microanatomy, electrophysiology, and mathematical modeling. Anatomical features of the transitional zone may explain its vulnerability to mechanical stress, which causes nerve fiber degeneration secondary to vascular compression. Demyelination, a key component of this degeneration, induces insulation failure and redistribution of ion channels. These biophysical changes give rise to pathogenic electrophysiological phenomena, including ectopic firing and ephaptic transmission. This pathophysiological evidence may account for characteristic clinical symptoms, such as the trigger zone in TN patients and the LSR in HFS patients. These chronic abnormal discharges from the peripheral segment induce pathophysiological alterations in the CNS. While various treatments have been established based on pathophysiology, novel drug discovery approaches targeting remyelination or restoration of ion channel dynamics to recover axonal transmission in degenerative nerve fibers are anticipated. Furthermore, neuromodulation therapy targeting the CNS via direct electrical intervention represents a possible direction for future investigation. Improved integrative understanding of NVCS pathophysiology may facilitate development of novel treatments.

## Figures and Tables

**Table 1:** Methodological features, key findings, and clinical implications of mathematical models for ephaptic transmission.

Model	Key Feature, Assumption, and Limitation	Key Findings	Clinical and Pathophysiologic Implication
Cable Model (Section 5.2)	Models two parallel axons with identical diameters. Theoretical model; not directly validated in TN or HFS. Does not reflect structural changes with demyelination of axons.	Formulation of ephaptic transmission intensity using the coupling parameter α.	Theoretical basis for demyelination-induced breakdown of axonal isolation.
Fiber Bundle Conduction Simulation (Section 5.3)	Models a multi-fiber bundle containing up to 500 fibers. Incorporates the nodes of Ranvier. Simulates focal demyelination. Perfectly parallel, axisymmetric, and identically sized fibers with nodal ion channels and passive myelin sheaths. The mathematical model is based on experimentally structured Cable Theory, but concepts found in simulation are not yet verified experimentally.	Conduction block induced by demyelination. Synchronization of spikes among parallel fibers. Elicitation of “spurious spikes” in unstimulated fibers. Conduction restoration in demyelinated fibers facilitated by intact fibers.	Suggests the cross-modal activation in TN (tactile fibers triggering nociceptive fibers) and HFS (motor fibers triggering adjacent branches). Simulates an endogenous electrophysiological compensatory mechanism against demyelination.
Quasi-static Electric Dipole Model (Section 5.4)	Models voltage-gated ion channels as electric dipoles. Experimentally unvalidated concept.	Generation of induced electric fields originating from the nodes of Ranvier. ”Zigzag conduction” through the induction of membrane potentials in adjacent fibers.	Attenuation of the induced electric field due to demyelination. Ectopic firing driven by the high packing density of demyelinated fibers.

## Data Availability

Original contributions presented in this study are included in the article. Further inquiries should be directed to the corresponding author.

## Abbreviations

The following abbreviations are used in this manuscript:

NVCS	Neurovascular compression syndromes
TN	Trigeminal neuralgia
HFS	Hemifacial spasm
MVD	Microvascular decompression
CNS	Central nervous system
PNS	Peripheral nervous system
MS	Multiple Sclerosis
idTNC	intradural trigeminal nerve compression
IL-6	Interleukin-6
LSR	Lateral spread response
S1	Primary somatosensory cortex
tDCS	Transcranial direct current stimulation
M1	Primary motor cortex
FMN	Facial motor nucleus
AMR	Abnormal muscle response

## References

[1] Shi X., Zhang X., Xu L., Xu Z. (2022). Neurovascular compression syndrome:Trigeminal neuralgia, hemifacial spasm, vestibular paroxysmia, glossopharyngeal neuralgia, four case reports and review of literature. Clin. Neurol. Neurosurg..

[2] Hannan C., Shoakazemi A., Quigley G. (2018). Microvascular Decompression for Trigeminal Neuralgia: A regional unit’s experience. Ulst. Med. J..

[3] Lu A.Y., Yeung J.T., Gerrard J.L., Michaelides E.M., Sekula R.F., Bulsara K.R. (2014). Hemifacial Spasm and Neurovascular Compression. Sci. World J..

[4] Patel S.K., Markosian C., Gault F.O., Liu J.K. (2020). The historical evolution of microvascular decompression for trigeminal neuralgia: From Dandy’s discovery to Jannetta’s legacy. Acta Neurochir..

[5] Dandy W.E. (1934). Concerning the cause of trigeminal neuralgia. Am. J. Surg..

[6] Jannetta P.J. (1970). Microsurgical exploration and decompression of the facial nerve in hemifacial spasm. Curr. Top. Surg. Res..

[7] De Ridder D., Møller A., Verlooy J., Cornelissen M., De Ridder L. (2002). Is the Root Entry/Exit Zone Important in Microvascular Compression Syndromes?. Neurosurgery.

[8] Tesařová M., Peterková L., Šťastná M., Kolář M., Lacina L., Smetana K., Hynek R., Betka J., Vlasák A., Lukeš P. (2022). Tumor Biology and Microenvironment of Vestibular Schwannoma-Relation to Tumor Growth and Hearing Loss. Biomedicines.

[9] Quanchareonsap W., Jariyakosol S., Apinyawasisuk S., Agthong S. (2022). Microanatomy of the central myelin portion and transitional zone of the oculomotor and abducens nerves. Folia Morphol..

[10] Haller S., Etienne L., Kövari E., Varoquaux A.D., Urbach H., Becker M. (2016). Imaging Neurovascular Compression Syndromes: Trigeminal Neuralgia, Hemifacial Spasm, Vestibular Paroxysmia, and Glossopharyngeal Neuralgia. AJNR Am. J. Neuroradiol..

[11] Hughes M.A., Frederickson A.M., Branstetter B.F., Zhu X., Sekula R.F. (2016). MRI of the Trigeminal Nerve in Patients with Trigeminal Neuralgia Secondary to Vascular Compression. AJR Am. J. Roentgenol..

[12] Yee G.T., Yoo C.J., Han S.R., Choi C.Y. (2014). Microanatomy and Histological Features of Central Myelin in the Root Exit Zone of Facial Nerve. J. Korean Neurosurg. Soc..

[13] Jia D.Z., Li G. (2010). Bioresonance hypothesis: A new mechanism on the pathogenesis of trigeminal neuralgia. Med. Hypotheses.

[14] Hilton D.A., Love S., Gradidge T., Coakham H.B. (1994). Pathological findings associated with trigeminal neuralgia caused by vascular compression. Neurosurgery.

[15] Devor M., Govrin-Lippmann R., Rappaport Z.H. (2002). Mechanism of trigeminal neuralgia: An ultrastructural analysis of trigeminal root specimens obtained during microvascular decompression surgery. J. Neurosurg..

[16] Headache Classification Committee of the International Headache Society (IHS) (2018). The International Classification of Headache Disorders, 3rd edition. Cephalalgia.

[17] Lee A., McCartney S., Burbidge C., Raslan A.M., Burchiel K.J. (2014). Trigeminal neuralgia occurs and recurs in the absence of neurovascular compression. J. Neurosurg..

[18] Ishikawa M., Nishi S., Aoki T., Takase T., Wada E., Ohwaki H., Katsuki T., Fukuda H. (2002). Operative findings in cases of trigeminal neuralgia without vascular compression: Proposal of a different mechanism. J. Clin. Neurosci..

[19] Kimura T., Sameshima T., Morita A. (2012). Trigeminal neuralgia caused by a fibrous ring around the nerve. J. Neurosurg..

[20] Yoshida S., Tsuchiya T., Hanakita S., Oya S. (2026). Potential Role of Turbulent Cerebrospinal Fluid Flow in Type 1 Trigeminal Neuralgia Without Neurovascular Compression: Insights from a Cerebrospinal Fluid Dynamics Study. Neurosurgery.

[21] Ritchie J.M., Rang H.P., Pellegrino R. (1981). Sodium and potassium channels in demyelinated and remyelinated mammalian nerve. Nature.

[22] Moll C., Mourre C., Lazdunski M., Ulrich J. (1991). Increase of sodium channels in demyelinated lesions of multiple sclerosis. Brain Res..

[23] Craner M.J., Newcombe J., Black J.A., Hartle C., Cuzner M.L., Waxman S.G. (2004). Molecular changes in neurons in multiple sclerosis: Altered axonal expression of Nav1.2 and Nav1.6 sodium channels and Na+/Ca2+ exchanger. Proc. Natl. Acad. Sci. USA.

[24] Fields R.D. (2014). Myelin—More than Insulation. Science.

[25] Lubetzki C., Stankoff B. (2014). Demyelination in multiple sclerosis. Handb. Clin. Neurol..

[26] Shi R., Sun W.A. (2011). Potassium channel blockers as an effective treatment to restore impulse conduction in injured axons. Neurosci. Bull..

[27] Felts P.A., Kapoor R., Smith K.J. (1995). A mechanism for ectopic firing in central demyelinated axons. Brain.

[28] Abdulrahim M.W., Jiang C., He L., Zhong J. (2026). A mouse model of classical trigeminal neuralgia via intradural compression of the trigeminal nerve. J. Headache Pain.

[29] Mustafá E.R., Gambeta E., Stringer R.N., Souza I.A., Zamponi G.W., Weiss N. (2022). Electrophysiological and computational analysis of Cav3.2 channel variants associated with familial trigeminal neuralgia. Mol. Brain.

[30] Liao X., Luo Z., Huang F., Wang Y., Zeng Z., Liao W., Ou Y., Wu X., Wang F., Luo D. (2025). Trigeminal nerve root compression induced neuroinflammatory response promotes mechanical allodynia through the CGRP/SP-Piezo2 axis via Ca2+ signaling. Cell. Mol. Biol. Lett..

[31] Liu M.X., Li Y., Zhong J., Xia L., Dou N.N. (2021). The effect of IL-6/Piezo2 on the trigeminal neuropathic pain. Aging.

[32] Katz B., Schmitt O.H. (1940). Electric interaction between two adjacent nerve fibers. J. Physiol..

[33] Sheheitli H., Jirsa V.K. (2020). A mathematical model of ephaptic interactions in neuronal fiber pathways: Could there be more than transmission along the tracts?. Netw. Neurosci..

[34] Hodgkin A.L. (1937). Evidence for electrical transmission in nerve: Part I. J. Physiol..

[35] Reutskiy S., Rossoni E., Tirozzi B. (2003). Conduction in bundles of demyelinated nerve fibers: Computer simulation. Biol. Cybern..

[36] Frankenhaeuser B., Huxley A.F. (1964). The action potential in the myelinated nerve fiber of Xenopus Laevis as computed on the basis of voltage clamp data. J. Physiol..

[37] Schmidt H., Knösche T.R. (2022). Modelling the effect of ephaptic coupling on spike propagation in peripheral nerve fibres. Biol. Cybern..

[38] Guo Y.M., Ong C.K. (2024). Possible mechanism of action potential propagation mediated by ephaptic field: A novel assumption of understanding nerve interaction and ephaptic coupling. Heliyon.

[39] Di Stefano G., Maarbjerg S., Nurmikko T., Truini A., Cruccu G. (2018). Triggering trigeminal neuralgia. Cephalalgia.

[40] Grasso G., Landi A., Alafaci C. (2016). A Novel Pathophysiological Mechanism Contributing to Trigeminal Neuralgia. Mol. Med..

[41] DaSilva A.F., DosSantos M.F. (2012). The role of sensory fiber demography in trigeminal and postherpetic neuralgias. J. Dent. Res..

[42] Ding Y.Q., Qi J.G. (2022). Sensory root demyelination: Transforming touch into pain. Glia.

[43] Kim M., Park S.K., Kubota Y., Lee S., Park K., Kong D.S. (2022). Applying a deep convolutional neural network to monitor the lateral spread response during microvascular surgery for hemifacial spasm. PLoS ONE.

[44] Kameyama S., Masuda H., Shirozu H., Ito Y., Sonoda M., Kimura J. (2016). Ephaptic transmission is the origin of the abnormal muscle response seen in hemifacial spasm. Clin. Neurophysiol..

[45] Khodashenas M., Baghdadi G., Towhidkhah F. (2019). A modified Hodgkin-Huxley model to show the effect of motor cortex stimulation on the trigeminal neuralgia network. J. Math. Neurosci..

[46] Moller A.R. (1987). Hemifacial spasm: Ephaptic transmission or hyperexcitability of the facial motor nucleus?. Exp. Neurol..

[47] Joo B.E. (2023). Research on the Pathogenesis of Hemifacial Spasm through Electrophysiological Studies. J. Electrodiagn. Neuromuscul. Dis..

[48] da Silva Martins W.C., Rodrigues M.R., de Oliveira L.B., Santos G.P., de Sousa S.G., de Sousa D.A.J., de Oliveira F.M., da Silva N.I.B., de Araujo M.G.L. (2017). Tenth case of bilateral hemifacial spasm treated by microvascular decompression: Review of the pathophysiology. Surg. Neurol. Int..

[49] Rana M.H., Khan A.A.G., Khalid M.I., Ishfaq M., Javali M.A., Baig F.A.H., Kota M.Z., Khader M.A., Hameed M.S., Shaik S. (2023). Therapeutic Approach for Trigeminal Neuralgia: A Systematic Review. Biomedicines.

[50] Song H.G., Nahm F.S. (2020). Oxcarbazepine for trigeminal neuralgia may induce lower extremity weakness: A case report. World J. Clin. Cases.

[51] Sun G.C., Chang Y.F., Wang M.W. (2006). Kinetic changes and modulation by carbamazepine on voltage-gated sodium channels in rat CA1 neurons after epilepsy. Acta Pharmacol. Sin..

[52] Jo S., Bean B.P. (2014). Sidedness of Carbamazepine Accessibility to Voltage-Gated Sodium Channels. Mol. Pharmacol..

[53] Pineda-Farias J.B., Barragán-Iglesias P., Granados-Soto V. (2021). Mechanisms Underlying the Selective Therapeutic Efficacy of Carbamazepine for Attenuation of Trigeminal Nerve Injury Pain. J. Neurosci..

[54] Jesuthasan A., Natalwala A., Davagnanam I., Saifee T., Zrinzo L. (2025). Hemifacial spasm: An update on pathophysiology, investigations and management. J. Neurol..

[55] Zhong J., Zhu J., Sun H., Dou N.N., Wang Y.N., Ying T.T., Xia L., Liu M.X., Tao B.B., Li S.T. (2014). Microvascular decompression surgery: Surgical principles and technical nuances based on 4000 cases. Neurol. Res..

[56] Jiang C., Xu W., Dai Y., Lu T., Jin W., Liang W. (2017). Early permanent disappearance of abnormal muscle response during microvascular decompression for hemifacial spasm: A retrospective clinical study. Neurosurg. Rev..

[57] Lee M.H., Lee J.A., Park K. (2018). Different Roles of Microvascular Decompression in Hemifacial Spasm and Trigeminal Neuralgia. J. Neurol. Surg. B Skull Base.

[58] Zhang Z., Wang F., Yu F., Kwok S.C., Yin J. (2022). Delayed pain relief in patients with trigeminal neuralgia following microvascular decompression: A single-central retrospective study. Front. Neurol..

[59] Wang D.D., Raygor K.P., Cage T.A., Ward M.M., Westcott S., Barbaro N.M., Chang E.F. (2018). Prospective comparison of long-term pain relief rates after first-time microvascular decompression and stereotactic radiosurgery for trigeminal neuralgia. J. Neurosurg..

[60] Al Menabbawy A., El Refaee E., Elwy R., Salem A.A., Lehmann S., Vollmer M. (2022). A multivariable prediction model for recovery patterns and time course of symptoms improvement in hemifacial spasm following microvascular decompression. Acta Neurochir..

[61] Siddiqi I., Brazdzionis J., Hough J.M., Reier L., Marino M., Ko K., Schiraldi M., Cortez V., Miulli D.E. (2024). Evaluating Changes in Pulsatile Flow with Endovascular Stents in an In Vitro Blood Vessel Model: Potential Implications for the Future Management of Neurovascular Compression Syndromes. Cureus.

[62] Leandri M., Eldridge P., Miles J. (1998). Recovery of nerve conduction following microvascular decompression for trigeminal neuralgia. Neurology.

[63] Love S., Hilton D.A., Coakham H.B. (1998). Central Demyelination of the Vth Nerve Root in Trigeminal Neuralgia Associated with Vascular Compression. Brain Pathol..

[64] Nielsen V.K. (1985). Electrophysiology of the facial nerve in hemifacial spasm: Ectopic/ephaptic excitation. Muscle Nerve.

[65] Sultan H., Sabahi M., Gholamshahi H., Albakr A., Adada B., Borghei-Razavi H. (2025). Long-term outcomes of microvascular decompression for trigeminal neuralgia in multiple sclerosis: A systematic review and meta-analysis. J. Neurosurg..

[66] Filimonova E.A., Pashkov A.A., Yarnykh V.L., Martirosyan A.V., Moysak G.I., Rzaev J.A. (2025). Assessment of Trigeminal Nerve Root Demyelination in Patients with Primary Trigeminal Neuralgia Using Macromolecular Proton Fraction Imaging. AJNR Am. J. Neuroradiol..

[67] Loayza R., Tan K.S., Sekula R.F. (2023). Outcome after microvascular decompression for trigeminal neuralgia in a single center-relation to sex and severity of neurovascular conflict. Acta Neurochir..

[68] Li M.W., Jiang C., Zhong J. (2020). Clinical Research on Delayed Cure after Microvascular Decompression for Hemifacial Spasm. J. Neurol. Surg. A Cent. Eur. Neurosurg..

[69] Deng Z., Liu R., Liu Y., Wang Z., Yu Y., Zhang L. (2019). Factors That May Affect Delayed Relief of Trigeminal Neuralgia After Microneurosurgery and The Long-Term Outcomes Associated with Delayed Relief. J. Pain Res..

[70] Tuleasca C., Régis J., Sahgal A., De Salles A., Hayashi M., Ma L., Martínez-Álvarez R., Paddick I., Ryu S., Slotman B.J. (2019). Stereotactic radiosurgery for trigeminal neuralgia: A systematic review. J. Neurosurg..

[71] Lee S., Lee J.I. (2022). Gamma Knife Radiosurgery for Trigeminal Neuralgia: Review and Update. J. Korean Neurosurg. Soc..

[72] Lindquist C. (1995). Gamma Knife Radiosurgery. Semin. Radiat. Oncol..

[73] Nurmikko T.J., Eldridge P.R. (2001). Trigeminal neuralgia-pathophysiology, diagnosis and current treatment. Br. J. Anaesth..

[74] Park C.K., Lim S.H., Park K. (2023). Clinical Application of Botulinum Toxin for Hemifacial Spasm. Life.

[75] Tereshko Y., Dal Bello S., Lettieri C., Belgrado E., Gigli G.L., Merlino G., Valente M. (2024). Botulinum Toxin Type A for Trigeminal Neuralgia: A Comprehensive Literature Review. Toxins.

